# The dietary calcium-to-magnesium ratio and its association with glycemic control in type 2 diabetes: a cross-sectional study

**DOI:** 10.1007/s10534-026-00834-6

**Published:** 2026-07-20

**Authors:** Fabianne Nascimento Andrade, Matheus Menezes-Santos, Andréa Costa Goes, Ramara Kadija Fonseca Santos, Silvânio Silvério Lopes da Costa, Samir Hipólito dos Santos, Vivianne de Sousa Rocha, Ana Mara de Oliveira e Silva, Liliane Viana Pires

**Affiliations:** 1 Nutritional Biochemistry Laboratory, Department of Nutrition, Center for Biological and Health Sciences, Federal University of Sergipe, São Cristóvão, Sergipe 49107-230 Brazil; 2 Nutrition Sciences Post-Graduation Program, Department of Nutrition, Center for Biological and Health Sciences, Federal University of Sergipe, São Cristóvão, Sergipe Brazil; 3 Health Sciences Post-Graduation Program, Center for Biological and Health Sciences, Federal University of Sergipe, São Cristóvão, Sergipe Brazil; 4 Nucleus of Competence in Oil, Gas and Biofuels – NUPEG, Federal University of Sergipe, São Cristóvão, Sergipe Brazil; 5 Chemistry Post-Graduation Program, Chemistry Institute, Federal University of Bahia, Salvador, Bahia Brazil; 6 Department of Nutrition, Federal University of Sergipe, Lagarto, Sergipe Brazil

**Keywords:** Insulin Resistance, Glycemic Control, Magnesium, Calcium, Magnesium Deficiency, Calcium to magnesium ratio

## Abstract

Inadequate magnesium (Mg) and calcium (Ca) intake has been associated with impaired glucose homeostasis in type 2 diabetes mellitus (T2DM). An imbalanced dietary Ca/Mg ratio may affect the concentrations of these nutrients and contribute to poor disease control. This study investigated the association between the dietary Ca/Mg ratio, serum Ca and Mg levels, and glycemic biomarkers in individuals with T2DM. A cross-sectional observational study was conducted in adults with T2DM. Body weight, height, waist circumference, blood pressure, body fat percentage, and body mass index were assessed. Dietary Ca and Mg intake was evaluated, and the Ca/Mg ratio was calculated. Plasma Ca and Mg were measured by inductively-coupled plasma optical emission spectrometry. Fasting glucose, glycated hemoglobin, C-peptide, parathyroid hormone, and indices of insulin resistance (HOMA-IR), β-cell function (HOMA-%B), and insulin sensitivity (HOMA-%S) were assessed. Participants were stratified according to dietary Ca/Mg ratio (≤ 2 and > 2). Mann–Whitney or Student’s t tests, Pearson’s chi-square test, and linear regression analyses were performed (p < 0.05). Of 115 individuals evaluated, 74% were females, with a median age of 49 years and median T2DM duration of 6 years. All participants had inadequate Mg intake (median dietary Ca/Mg ratio: 2.3). A lower dietary Ca/Mg ratio was correlated with higher HOMA-%S (p = 0.049), whereas a higher ratio correlated with higher HOMA-IR (p = 0.05) and C-peptide levels (p = 0.008). Furthermore, a higher dietary Ca/Mg ratio was associated with lower insulin sensitivity (β = − 4.588, p = 0.038). An imbalance between Mg and Ca levels was associated with greater insulin resistance in individuals with T2DM.

## Introduction

Diabetes mellitus (DM) is a chronic noncommunicable disease with increasing global prevalence, currently affecting approximately 589 million adults worldwide. In 2024, Brazil ranked sixth globally in the number of DM cases, underscoring the substantial public health burden of the disease (International Diabetes Federation 2025).

This disease is characterized by metabolic disturbances resulting from impaired regulation of blood glucose levels (American Diabetes Association, 2013). Type 2 diabetes mellitus (T2DM), the most prevalent form, has a multifactorial origin involving strong genetic and environmental contributions to its development (Rodacki et al. 2023). Unhealthy lifestyle behaviors, including physical inactivity and inadequate food consumption, are important risk factors for the development of T2DM (Davies et al. 2022).

Insulin resistance is a central pathophysiological mechanism in the development of T2DM. In response to insulin resistance, glycemic homeostasis is initially maintained through compensatory hyperinsulinemia; however, persistent metabolic stress ultimately leads to progressive β-cell dysfunction (Nolan and Prentki 2019). However, β-cell hyperreactivity and dysfunction may, in some cases, precede the onset of insulin resistance in T2DM (Nolan and Prentki 2019). Chronic hyperglycemia in DM is associated with long-term damage, dysfunction, and failure of multiple organs, leading to substantial morbidity and reduced quality of life in affected individuals (American Diabetes Association, 2013).

Scientific evidence indicates that micronutrients play crucial roles in the development and management of DM (Wu et al. 2017; Mears 2004; Zaccardi et al. 2015; Ojuka et al. 2002; Pittas et al. 2007; Rooney et al. 2016). Magnesium (Mg) acts as a cofactor and enzymatic activator in multiple pathways involved in glucose and energy metabolism (Kostov 2019). Deficiency of this mineral may impair the phosphorylation of key components of the insulin secretion signaling pathway and disrupt the different phases of insulin release, thereby contributing to hyperglycemia (Kostov 2019). Inadequate Mg status has also been associated with insulin resistance (Bavani et al. 2021) and with an increased risk of T2DM (Wu et al. 2017; Mears 2004; Zaccardi et al. 2015; Ojuka et al. 2002; Pittas et al. 2007; Rooney et al. 2016; Kostov 2019; Fang et al. 2016).

Similarly, calcium (Ca) plays an essential role in insulin signaling, including the phosphorylation of the insulin receptor (Mears 2004). In addition, insulin secretion is regulated by processes dependent on intracellular Ca concentrations within pancreatic β-cells (Zaccardi et al. 2015). Elevated cytosolic Ca concentrations may influence glucose uptake in tissues such as the liver, adipose tissue, and skeletal muscle (Ojuka et al. 2002; Pittas et al. 2007). Furthermore, observational studies have suggested that higher serum Ca concentrations are associated with an increased risk of T2DM (Rooney et al. 2016; Lorenzo et al. 2014), underscoring the importance of tightly regulated Ca homeostasis for optimal insulin signal transduction (Zemel 1998).

Mg acts as a physiological Ca antagonist, as it competes for protein-binding sites and Ca transport channels; moreover, high Ca intake may reduce Mg absorption (de Baaij et al. 2015). A previous study reported lower serum Mg concentrations in women without T2DM who had a dietary Ca/Mg ratio of 2:1, whereas women with T2DM exhibited a dietary Ca/Mg ratio of 2:2 (Kocyigit et al. 2023).

Although there is no established consensus regarding the optimal dietary Ca/Mg ratio, some studies have suggested that it should be maintained close to 2 (Kocyigit et al. 2023; Durlach 1989). Associations between serum Ca and Mg concentrations and the Ca/Mg ratio have been reported in patients with coronary artery disease (Li et al., 2020) and in individuals undergoing dialysis (Sato et al. 2017); however, evidence regarding this ratio in patients with T2DM remains limited.

However, important knowledge gaps remain regarding the dietary Ca/Mg ratio, the interactions between these minerals in T2DM, factors affecting their concentrations, and their potential contributions to disease management and progression. Therefore, this study aimed to investigate the association between the dietary Ca/Mg ratio, serum Ca and Mg levels, and glycemic biomarkers in individuals with T2DM.

## Materials and methods

### Study design and participants

We conducted an observational cross-sectional study involving 115 individuals with T2DM, aged 19 to 59 years, of both sexes, diagnosed with T2DM and followed in Primary Health Care services in the state of Sergipe, Brazil. The participants were voluntarily recruited from Basic Health Units after the study was publicized via digital media and within the community. Exclusion criteria were smoking, alcohol consumption, and the use of food supplements or medications such as corticosteroids in the three months before data collection; pregnant women and individuals with chronic or acute inflammatory conditions (rheumatoid arthritis, cancer, chronic kidney disease, thyroid dysfunction, and self-reported infections); and acute inflammatory conditions such as flu and urinary tract infection.

The statistical power of the sample was calculated post hoc based on the observed effect size in the multivariate linear regression analysis between the dietary Ca/Mg ratio and insulin sensitivity, as assessed by the homeostatic model assessment for insulin sensitivity (HOMA-%S), in the 115 study participants in the study. The analysis yielded a regression coefficient (β) of −4.605 (95% confidence interval: −8.848 to 0.261), with an alpha level of 0.05, statistical power of 0.99, and a critical z-value of 1.64.

### Data collection

Semi-structured questionnaires were used to collect socioeconomic and clinical information (time since diagnosis, use of oral antidiabetics, presence of comorbidities, and physical activity). Dietary intake data were also obtained. Anthropometric measurements, including body weight, height, and waist circumference (WC), and body composition, were performed to characterize the participants. Blood pressure was measured. Additionally, venous blood samples were collected to assess plasma concentrations of Mg and Ca and markers of glycemic control, including percentage of glycated hemoglobin (%HbA1c), and serum fasting glucose, insulin, and C-peptide. Parathyroid hormone (PTH) levels were also measured.

All participants provided written informed consent. The study was conducted in accordance with the principles of the Declaration of Helsinki and was approved by the Research Ethics Committee of the Federal University of Sergipe (no. 3.012.056).

### Assessment of dietary Mg and Ca intake

To assess the dietary intake of Ca and Mg, as well as energy and macronutrients, three 24-h dietary recalls were conducted on non-consecutive days. The 24-h recalls were based on the Multiple Pass Method, which involves five steps: reporting all foods consumed by the participant without interruptions; reviewing all the foods reported with questions about the consumption of food items that may be forgotten; recording the time and name of each meal; detailing the food items, culinary preparations, added ingredients, brands, and flavors; and performing a final review of the foods consumed (Raper et al. 2004).

Nutwin software, developed by the Health Informatics Department of the Federal University of São Paulo, was used to quantify the intake of energy, protein, carbohydrates, lipids, Ca, and Mg from dietary sources. The dietary Ca/Mg ratio was calculated by dividing Ca intake (mg/day) by Mg intake (mg/day). According to Kocyigit et al. (2023) and Durlach et al. (1989), a ratio of ≤ 2 was considered adequate.

### Assessment of anthropometrics measurements and body composition

Weight, height, and WC were measured using techniques standardized by the World Health Organization (WHO) and Food and Nutrition Surveillance System (SISVAN) (WHO 2000; Brasil 2022). Body Mass Index (BMI) was calculated as the ratio of weight (kg) to the square of height (m) and classified according to the WHO criteria (WHO 2000), using the following cut-off points: < 18.5 kg/m^2^ (underweight), 18.5–24.9 kg/m^2^ (normal weight), 25.0–29.9 kg/m^2^ (overweight), and ≥ 30.0 kg/m^2^ (obesity).

Body composition was assessed using bioelectrical impedance analysis (BIA) (Bioimpedance Analyzer BIA 310; Biodynamics Corporation, Shoreline, WA, USA). The participants were instructed to avoid caffeinated beverages, as well as to refrain from intense physical activity for 24 h before the test. At the time of assessment, the participants were asked to empty their bladders. Women who were menstruating underwent test rescheduling. Percentage body fat was classified according to the reference values proposed by Lohman et al. (1992), with individuals > 30% classified as overweight.

### Measurement of blood pressure

The participants were instructed to rest for 3–5 min before undergoing blood pressure measurements. It was also ensured that they did not have a full bladder, had not performed physical activity within 60 min before the measurement, and had not used tobacco within 30 min prior to the assessment. Blood pressure was measured and classified according to the recommendations of the Brazilian Society of Cardiology (Malachias et al. 2016).

### Blood collection

The participants were instructed to fast overnight for 10 h. Blood samples (15 mL) were collected using sterile disposable syringes and distributed into different tubes: tubes containing the anticoagulant ethylenediaminetetraacetic acid (EDTA; Vacuette, Greiner Bio-One, Brazil) for %HbA1c determination, dry tubes (Vacuette, Greiner Bio-One, Brazil) for serum collection to analyze glycemic and lipid profile markers and PTH levels. Demineralized polypropylene tubes containing 30% sodium citrate anticoagulant were used to obtain plasma samples for Mg and Ca analyses. Serum and plasma were separated from whole blood by centrifugation at 3000 rpm for 15 min at 4 °C. Samples were then stored at − 80 °C until analysis.

To prevent contamination, all materials used for mineral analysis (including glassware, plastics, pipette tips, and tubes) were demineralized in a 20% nitric acid bath for at least 12 h and rinsed 10 times with deionized water (Boa Morte et al. 2012). Sterile materials were used for all other analyses.

### Measurement of Mg and Ca levels in plasma

Plasma Mg (λ = 279.553 nm) and Ca (λ = 211.276 nm) concentrations were determined by inductively coupled plasma optical emission spectrometry (ICP-OES) using an axially viewed instrument (model ES-720, Agilent Technologies, Mulgrave, Australia). Initially, a solution containing 0.5 mL of plasma, 2 mL of concentrated nitric acid, and 3 mL of ultrapure water (resistivity 18.2 MΩ·cm; Gehaka, Master System All, São Paulo, SP, Brazil) was subjected to acid digestion in a temperature-controlled block digester. The digestion was carried out in capped polytetrafluoroethylene (PTFE) vessels (model TE007-A, TECNAL, São Paulo, SP, Brazil) for 2 h at 150 °C. After digestion, the samples were diluted with ultrapure water to a final volume of 15 mL. Reagent blanks were prepared for each digestion batch under the same analytical conditions described by Castro et al. (2009). All samples were prepared in triplicate, and three replicate measurements were obtained for each preparation.

Method precision and accuracy were evaluated using spike-and-recovery assays at four concentration levels (5, 50, 500, and 1000 mg/L) for each element analyzed, selected to represent the expected concentration ranges of these minerals in plasma samples.

Precision was < 10%, and accuracy ranged from 87 to 119%, indicating that the method is suitable for the determination of these elements in human plasma (Santos et al. 2021). Additionally, a certified reference material (Seronorm™ Trace Elements Serum; Sero AS, Billingstad, Norway) was reanalyzed along with a reagent blank and an intermediate calibration standard after every 10 samples.

The limits of detection (LOD) and quantification (LOQ) for Mg were 0.001 and 0.005 mg/dL, respectively, whereas those for Ca were 0.774 and 2.555 mg/L, respectively, supporting the analytical reliability of the method (Silva et al. 2002; Skoog et al. 2002). Results were initially expressed in mg/L and subsequently converted to the units corresponding to the reference cut-off values: Mg > 2.07 mg/dL (Rosanoff et al. 2022) and Ca > 41.9 mg/L (Deng et al. 2008).

### Biochemical assessment of hormonal and metabolic parameters

The %HbA1c was analyzed using an immunoturbidimetric inhibition assay, and fasting plasma glucose was measured using an enzymatic colorimetric method with commercial kits (Labtest®, Lagoa Santa, Minas Gerais, Brazil). Individuals with %HbA1c ≥ 7% and fasting plasma glucose ≥ 130 mg/dL were classified as having poor glycemic control (ElSayed et al. 2023). Serum C-peptide and insulin concentrations were measured by chemiluminescence using the Architect i1000SR immunoassay analyzer (Abbott®, Abbott Park, IL, USA) with commercially available assay kits (Abbott®, Abbott Park, IL, USA).

The C-peptide values were used because they are co-secreted with insulin in equimolar amounts and are not affected by exogenous insulin administration. Therefore, C-peptide can be used to estimate endogenous insulin secretion (Jones and Hattersley 2013). Insulin resistance was assessed using the Homeostasis Model Assessment for Insulin Resistance (HOMA-IR) index, calculated from fasting serum C-peptide and glucose concentrations with the HOMA calculator developed by the University of Oxford, UK. The calculation was based on the updated homeostasis model (Levy et al. 1998). Participants with HOMA-IR values > 2.71 were classified as insulin resistant (Geloneze et al. 2009). Additionally, β-cell function and insulin sensitivity were assessed by calculating HOMA-%B and HOMA-%S, respectively, to further characterize the study population.

In addition, serum 25-hydroxyvitamin D [25(OH)D] concentrations were measured by immunoturbidimetry. PTH concentrations were measured by chemiluminescent immunoassay (DXI800 analyzer, Beckman Coulter®, Brea, CA, USA) with commercially available kits.

### Statistical analysis

Data were analyzed using the Statistical Package for Social Sciences software, version 30.0. Initially, a descriptive analysis of the data was performed, with categorical variables presented as absolute and relative frequencies (n [%]) and continuous variables presented as means, standard deviations, medians, and interquartile ranges, according to the visual inspection of histograms and normality of the data analyzed using the Kolmogorov–Smirnov test. Variables presenting normal distribution were described as mean ± standard deviation, whereas non-normally distributed variables were expressed as median and interquartile range.

Energy and nutrient intake (lipids, proteins, carbohydrates, Ca, and Mg) was assessed for intrapersonal variability using the Multiple Source Method (MSM) (Harttig et al. 2011). The participants were divided into two groups based on their dietary Ca/Mg ratio (≤ 2 and > 2). According to the distribution of the data, comparisons between the groups were performed using the Mann–Whitney U test or Student’s t-test for independent samples, according to the normality of the data. The association between categorical variables (sex, glycemic control [fasting glucose and %HbA1c], and dietary Ca/Mg ratio) was determined using Pearson’s chi-square test when the assumption of expected frequencies (≥ 5 in at least 80% of cells) was satisfied or Fisher’s exact when this assumption was violated.

To evaluate the association between glycemic variables (dependent variables) and the dietary Ca/Mg ratio (independent variable), multivariable linear regression analyses were performed, adjusting for age (years), sex, duration of T2DM, percentage body fat, energy intake (kcal), 25(OH)D levels and use of oral antidiabetic and antihypertensive drugs. P values ≤ 0.05 were considered statistically significant.

## Results

A total of 115 individuals with T2DM were evaluated. The median age was 49 years, and the median duration since diagnosis was 6 years; 74% of participants were female. Most participants were using oral antidiabetic agents (86.1%), had overweight (37.7%) or obesity (44.7%), and presented with an elevated percentage of body fat (62.7%). Regular physical activity was reported by 46.1% of participants. Additionally, 86% of participants had elevated WC. The median systolic and diastolic blood pressures were 126 and 80 mmHg, respectively. All participants exhibited Mg deficiency, defined as a plasma Mg concentration < 2.07 mg/dL.

The median dietary Ca/Mg ratio was 2.3 (interquartile range: 1.3). Participants with a dietary Ca/Mg ratio > 2 had lower energy (p = 0.036) and protein intake (p < 0.001) compared with those with a dietary Ca/Mg ratio ≤ 2. Differences in Ca and Mg intake between the groups were expected, as these variables were used to calculate the Ca/Mg ratio. The remaining variables did not differ significantly according to the dietary Ca/Mg ratio. Clinical and anthropometric characteristics, biochemical markers of metabolic control, plasma Ca and Mg concentrations, and dietary intake data are presented in Table 1.

**Table 1:** Characterization of the study population according to the dietary Ca/Mg ratio

Variables	Total (n = 115)	Ca/Mg ratio ≤ 2 (n = 47)	Ca/Mg ratio > 2 (n = 68)	*P-*value*
Age, years	49.0 (10.0)	47.0 (8.0)	51.0 (9.7)	0.067^b^
Sex, n (%)				0.237^c^
Women	74 (64.3)	27 (57.4)	47 (69.1)	
Men	41 (35.7)	20 (42.6)	21 (30.9)	
Time of T2DM diagnosis, years	6.0 (9.0)	4.0 (9.0)	6.0 (10.0)	0.054^a^
Oral antidiabetics use, n (%)	99 (86.1)	39 (83.0)	60 (88.2)	0.428^c^
Physical exercise, n (%)	53 (46.1)	23 (48.9)	30 (44.1)	0.704^c^
SBP, mmHg^d^	126.0 (20.0)	124.0 (20.0)	128.0 (20.5)	0.854^a^
DBP, mmHg^d^	80.0 (13.0)	80.0 (12.0)	80.0 (14.5)	0.704^a^
Body mass index, kg/m^2^	29.7 (8.2)	29.0 (7.0)	29.8 (9.3)	0.397^a^
Body fat, %^e^	33.9 (± 8.0)	32.8 (± 8.2)	34.6 (± 7.8)	0.254^b^
Waist circumference, cm^e^	99.8 (± 13.7)	99.0 (± 13.8)	100.3 (± 16.7)	0.628^b^
Fasting serum glucose, mg/dL	153.0 (124.8)	155.0 (98.0)	150.9 (147.7)	0.724^a^
Glucose > 130 mg/dL, n (%)	68.0 (59.1)	30 (63.8)	38 (55.9)	0.444^c^
%HbA1c^f^	7.5 (3.4)	7.5 (2.7)	7.6 (3.9)	0.629^a^
> 7%, n (%)	67.0 (58.8)	27 (57.4)	40 (59.7)	0.848^c^
C-peptide, ng/mL	2.6 (1.4)	2.2 (1.6)	2.8 (1.5)	0.079^a^
HOMA-%B	60.2 (93.0)	49.1 (76.1)	72.3 (97.2)	0.381^a^
HOMA-%S	45.5 (± 21.1)	48.7 (± 21.0)	43.2 (± 21.0)	0.172^b^
HOMA-IR	2.2 (1.5)	2.1 (1.4)	2.2 (1.5)	0.160^a^
Parathormone, pg/mL^f^	33.0 (15.0)	30.0 (16.0)	34.0 (12.0)	0.076^a^
Plasmatic magnesium, mg/dL	0.5 (0.1)	0.5 (0.1)	0.6 (0.1)	0.772^a^
Plasmatic calcium, mg/L^f^	53.2 (35.0)	50.5 (36.5)	58.0 (32.6)	0.380^a^
25-hydroxyvitamin D, ng/mL^f^	31.8 (12.3)	31.2 (14.4)	32.6 (11.6)	0.566^a^
Energy, kcal/day	1527.4 (± 419.0)	1625.7 (± 388.9)	1459.5 (± 577.2)	**0.036**^b^
Dietary lipid, g/day	38.2 (21.1)	38.3 (17.4)	37.7 (23.2)	0.384^a^
Dietary protein, g/day	81.7 (± 14.0)	88.1 (± 13.6)	77.3 (± 12.6)	** < 0.001**^b^
Dietary protein, g/kg body weight/day	1.1 (± 0.3)	1.1 (± 0.3)	1.0 (± 0.3)	**0.010**^b^
Dietary carbohydrate, g/day	213.3 (± 62.2)	221.8 (± 71.5)	207.4 (± 64.8)	0.222^b^
Dietary magnesium, mg/day	180.6 (98.0)	214.0 (108.8)	173.5 (83.5)	** < 0.00**1^a^
Dietary calcium, mg/day	398.8 (254.5)	340.6 (152.0)	478.4 (218.2)	** < 0.001**^a^

Values are described as mean (± standard deviation), median (Interquartile Range), and frequencies (n [%]). Statistical significance was set at p < 0.05 was considered significant.

*T2DM* type 2 diabetes mellitus, *SBP* systolic blood pressure, *DBP* diastolic blood pressure *%HbA1c* percentage of glycated hemoglobin, *HOMA-%B* homeostatic model for assessing β-cell activity, *HOMA-%S* homeostatic model for assessing insulin sensitivity, *HOMA-IR* homeostatic model for assessing insulin resistance

*Mann–Whitney^a^ or T-Student tests^b^ for non-dependent samples, depending on the normality of the data. ^c^The Fisher’s exact or chi-square test for categorical variables. ^d^n = 113. ^e^n = 110. ^f^n = 114

Additionally, HOMA-%S was inversely correlated with the dietary Ca/Mg ratio (p = 0.049). Conversely, HOMA-IR and C-peptide concentrations were positively correlated with the dietary Ca/Mg ratio (p = 0.050 and p = 0.008, respectively) (Table 2).

**Table 2:** Correlations between dietary Ca/Mg ratio and clinical, anthropometric, biochemical, and dietary variables

Variables	Dietary Ca/Mg ratio	
	R	P-value*
Age (years)	0.183	**0.050**
Time of T2DM diagnosis	0.154	0.099
SBP (mmHg)	− 0.009	0.924
DBP (mmHg)	− 0.101	0.288
BMI (kg/m^2^)	0.068	0.473
WC (cm)	0.072	0.448
Body fat (%)	0.132	0.170
Seric glucose (mg/dL)	− 0.044	0.638
HbA1c (%)	0.041	0.666
C-peptide (ng/mL)	0.244	**0.008**
HOMA-%B	0.104	0.270
HOMA-%S	− 0.184	**0.049**
HOMA-IR	0.184	**0.050**
PTH (pg/mL)	0.117	0.215
Mg plasmatic (mg/dL)	− 0.045	0.635
Ca plasmatic (mg/dL)	− 0.006	0.952
25-hydroxyvitamin D (ng/mL)	0.037	0.692
Energy (kcal/day)	− 0.218	**0.019**
Dietary lipid (g/day)	− 0.075	0.427
Dietary protein (g/day)	0.320	** < 0.001**
Dietary carbohydrate (g/day)	− 0.129	0.169
Mg dietary (mg/day)	− 0.480	** < 0.001**
Ca dietary (mg/day)	0.464	** < 0.001**

Spearman’s non-parametric correlation; results presented by the correlation coefficient (r) and P-value < 0.05;

*statistically significant.

*T2DM* type 2 diabetes mellitus, *SBP* systolic blood pressure, *DBP* diastolic blood pressure, *%HbA1c* percentage of glycated hemoglobin, *HOMA-%B* homeostatic model for assessing β-cell activity, *HOMA-%S* homeostatic model for assessing insulin sensitivity, *HOMA-IR* homeostatic model for assessing insulin resistance, *Mg* magnesium, *Ca* calcium

Among the biomarkers related to glycemic control, only HOMA-%S was significantly associated with the dietary Ca/Mg ratio and exhibited a normal distribution; therefore, it was the only variable evaluated in linear regression analyses. The association between HOMA-%S (dependent variable) and the dietary Ca/Mg ratio (independent variable) was examined. It was observed that a higher dietary Ca/Mg ratio was associated with lower insulin sensitivity (HOMA-%S) (β = − 4.588, p = 0.038), independent of adjustment variables (age, sex, duration of T2DM, percentage body fat, energy intake, 25(OH)D levels, and use of oral antidiabetic and antihypertensive drugs) (Table 3).

**Table 3:** Association between HOMA-%S and dietary Ca/Mg ratio in individuals with type 2 diabetes mellitus

Model	HOMA-%S		
	β^a^	95% CI	*P*-value
Intake Ca/Mg ratio	− 4.588	− 8.919 to − 0.257	**0**.**038**
Age (years)	− 0.417	** − **0.975 to 0.140	0.141
Sex^1^	1.243	** − **11.377 to 13.863	0.845
Time of T2DM diagnosis	0.001	** − **0.014 to 0.016	0.855
Percentage body fat	0.707	** − **0.120 to 1.534	0.093
Energy (kcal/day)	** − **0.008	** − **0.018 to 0.003	0.155
25-hydroxyvitamin D (ng/mL)	0.411	** − **0.087 to 0.908	0.105
Use of oral antidiabetics drugs^1^	4.718	** − **7.375 to 16.810	0.441
Use of antihypertensive drugs^1^	0.184	** − **8.176 to 8.544	0.965

^a^Multivariate linear regression between HOMA-%S and Ca/Mg ratio, adjusted for age, sex, duration of diagnosis, percentage body fat, energy intake, 25-hydroxyvitamin D levels and use of oral antidiabetic and antihypertensive drugs. ^1^ Categorical data: sex, 1 female; 2 male. Use of oral antidiabetic and antihypertensive drugs: 0, no; 1, yes.

*HOMA-% S* homeostasis model assessment of insulin sensitivity, *Ca* Calcium, *Mg* Magnesium, *CI* Confidence Interval

## Discussion

We observed an association between a higher dietary Ca/Mg ratio and lower insulin sensitivity, suggesting that the balance between dietary Ca and Mg intake may be relevant to insulin sensitivity. It is known that the pathophysiology of T2DM involves multiple processes, including impaired insulin synthesis by pancreatic β-cells and decreased insulin sensitivity (Stumvoll et al. 2005).

Similar to the findings of this study, a case–control study reported that participants with T2DM had lower serum Mg concentrations and significantly lower HOMA-%S values than those without the disease (Moustafa 2016). Several cross-sectional studies have observed a higher prevalence of Mg deficiency in patients with T2DM, which is associated with impaired glycemic control and alterations in kidney function (Barragán et al. 2020; Wahid et al. 2017; Sales et al. 2011).

We observed that individuals with a dietary Ca/Mg ratio ≤ 2 consumed more calories, protein, and Mg, but less Ca, compared with those with a dietary Ca/Mg ratio > 2. Despite these differences in caloric and dietary protein intake between the groups, Mg and Ca intake remained below the recommendations established by the Dietary Reference Intakes (DRI). The amount of protein consumed per kilogram of body weight was adequate, which may be related to differences in dietary patterns and mineral intake. It is noteworthy that energy intake was included as an adjustment variable in the association model, due to its significant difference observed between the groups.

Furthermore, our study showed a positive correlation between the dietary Ca/Mg ratio and C-peptide levels as well as HOMA-IR, and a negative correlation with HOMA-%S. This finding supports the hypothesis that a greater imbalance between Ca and Mg may be related to higher insulin resistance. C-peptide reflects endogenous insulin secretion and is often used as an indicator of pancreatic β-cell activity (Vejrazkova et al. 2020). In a study by Kocyigit et al. (2023), the increased consumption of Mg-rich foods was associated with decreased HOMA-IR scores.

Hypomagnesemia and its relationship with insulin resistance have several hypothesized mechanisms. These include interactions with the tyrosine kinase activity of the insulin receptor and decreased insulin secretion and activity, which can decrease serum Mg levels (Kostov 2019; Barbagallo 2015). Notably, reduced Mg concentrations can lead to insulin resistance by increasing the intracellular Ca concentration, favoring the opening of L-type Ca channels, which are regulated by Mg-binding sites. When Mg binds to these sites, it blocks the influx of Ca into the cell; however, under conditions of low extracellular Mg concentration, this block is impaired, resulting in increased Ca entry, which can interfere with insulin signaling (Kostov 2019).

Mg deficiency in patients with T2DM appears to result from low mineral intake, decreased kidney reabsorption, and increased urinary excretion in the presence of hyperglycemia (Barbagallo 2015; Palathingal and Thomas 2020). According to the DRI, the estimated average requirement (EAR) of Mg for adults is 330–350 mg/day for men and 255–265 mg/day for women (IOM, 1997). Several studies conducted in individuals with T2DM have shown that these patients have a low intake of Mg (Sales et al. 2011; Huang et al. 2012), and some studies have shown that hypomagnesemia can present with hypocalcemia (Rude 1993; Kärkkäinen et al. 1997; Seelig 1964). A study of healthy adults with low Mg and Ca intake showed that a negative Ca balance was observed, which improved with increased Mg intake (Green et al. 2002). However, in our study, despite the participants having a low Ca intake, serum Ca concentrations remained adequate (> 41.9 mg/L) because of the tight regulation of Ca homeostasis, as reflected by the adequate PTH values (Wein and Kronenberg 2018).

In our study, we found no significant correlations between dietary Ca/Mg ratio and plasma Ca and Mg, fasting serum glucose, and %HbA1c levels. The relationship between dietary Ca/Mg ratio and glycemic markers has not yet been investigated in the T2DM population; however, reduced circulating Mg concentrations have frequently been associated with poor glycemic control. A case–control study of 150 individuals found a significant negative correlation between serum Mg, fasting plasma glucose, and %HbA1c in patients with T2DM and macrovascular complications compared with controls (Agrawal et al. 2011). In contrast, a meta-analysis of 24 randomized clinical trials reported that Mg supplementation significantly reduced fasting insulin concentrations and HOMA-IR scores in healthy individuals and those with T2DM, but no change was observed in %HbA1c (Zhao et al. 2019).

It is important to highlight that the heterogeneity of T2DM has recently gained increasing attention, particularly regarding efforts to subclassify the disease into distinct clinical subtypes. These include a subtype characterized by severe insulin deficiency (SIDD: Severe Insulin-Deficient Diabetes) and a subtype associated with increased insulin resistance (SIRD: Severe Insulin-Resistant Diabetes) (Ahlqvist et al. 2020). Given that a high dietary intake of Ca relative to that of Mg has been associated with greater insulin resistance (Kocyigit et al. 2023), this imbalance may be particularly relevant in individuals with the SIRD subtype. Therefore, the dietary Ca/Mg ratio may represent a relevant factor to consider in the management of T2DM and its subtypes.

Furthermore, no age difference was observed between the groups. Although a higher prevalence of women was reported in the group with a dietary Ca/Mg ratio > 2, no association of sex or age with the relationship between the Ca/Mg ratio and HOMA-%S was observed. However, an increased intake of Ca sources is encouraged in postmenopausal women, as a significant decline in estrogen synthesis is associated with low bone density, increased abdominal visceral, reduced skeletal muscle mass, and a lower basal metabolic rate, which may impair insulin sensitivity (Gallagher et al. 1987; Ko and Jung 2021). This may partially explain our findings. According to the DRI, the EAR and Recommended Dietary Allowance for women aged 51–70 years are 1,000 mg/day and 1,200 mg/day of Ca, respectively (IOM 2011); the two groups evaluated did not reach the Ca recommendations.

Although this study has a cross-sectional observational design, our findings highlight the potential relevance of Ca and Mg intake in the context of T2DM. Previous evidence indicates that both minerals play key roles in glucose metabolism, insulin secretion and action, and in the modulation of inflammatory processes and cellular homeostasis (Veronese et al. 2021; Klec et al. 2019). In this context, an adequate and balanced intake of Ca and Mg may represent a relevant nutritional target as an adjuvant strategy in the management of T2DM, although a causal relationship cannot be inferred from the current data. Plausible biological mechanisms underlying these associations include changes in insulin sensitivity, glucose transport, and mineral-dependent intracellular signaling (Barbagallo and Dominguez 2007; Gilon et al. 2014). Therefore, our findings should be interpreted with caution and highlight the need for longitudinal studies and randomized clinical trials to evaluate the effects of dietary or supplemental Ca and Mg on glycemic control and metabolic outcomes in individuals with T2DM.

However, our study advances the understanding of the relationship between the dietary Ca/Mg ratio and biomarkers of glucose metabolism, including HOMA indices, and is among the first to evaluate these associations. Furthermore, our findings suggest that the balance between dietary intake and body availability of these minerals may be related to insulin sensitivity and insulin resistance regulation, with possible implications for T2DM management. This study has some limitations, including the exclusive assessment of plasma Ca levels without evaluation of total body stores, which may limit the accuracy of determining Ca nutritional status.

## Conclusions

Our results showed that a higher dietary Ca/Mg ratio was independently associated with lower HOMA-%S. Additionally, the dietary Ca/Mg ratio was positively correlated with age, C-peptide levels, HOMA-IR, and protein intake. This association remained significant after adjustment for potential confounding variables, supporting the hypothesis that an imbalance between these minerals may be related to greater insulin resistance. Further studies are warranted to establish the optimal cut-off point for the dietary Ca/Mg ratio in individuals with T2DM and to inform more targeted nutritional and therapeutic strategies.

## Data Availability

No datasets were generated or analysed during the current study.
